# Evaluation of bleb characteristics after implantation of the EX-PRESS^™^ glaucoma filtration device

**Published:** 2012-01-04

**Authors:** Ashley E. Laing, Leonard K. Seibold, Jeffrey R. SooHoo, Malik Y. Kahook

**Affiliations:** University of Colorado, Department of Ophthalmology, Aurora, CO

## Abstract

**Purpose:**

To compare bleb survival and histology after implantation of the EX-PRESS™ glaucoma filtration device versus silicone tubes in a rabbit model of filtration surgery.

**Methods:**

Glaucoma filtration surgery was performed on one eye each of twelve New Zealand white rabbits. Eyes were randomized to implantation with the EX-PRESS™ filtration device (n=6) or a silicone tube (n=6). Bleb vascularity was evaluated at three and six weeks using a standard scale. At 6 weeks, eyes were enucleated and a histologic analysis was performed. Bleb survival was also recorded for the two groups.

**Results:**

Histologically, a thin capsule consisting of mild fibroblast proliferation associated with intercellular collagen was present around both implants. Both groups demonstrated a mild infiltration of plasma cells and polymorphonuclear leukocytes. Bleb vascularity was similar between both groups at three and six weeks post-operatively. Bleb survival between the two groups was not significantly different.

**Conclusions:**

Similar outcomes were noted after glaucoma filtration surgery using either silicone tubes or the EX-PRESS™ glaucoma filtration device in this rabbit model. Both implants appear to be relatively inert with little difference in biocompatibility and bleb survival.

## Introduction

Glaucoma is the second leading cause of blindness world-wide and is predicted to affect 79.6 million people by 2020 [1]. Glaucoma filtration surgery is commonly performed when other modalities, such as topical medications and laser trabeculoplasty, have failed to control intraocular pressure (IOP). Traditional implantable aqueous humor drainage devices, such as the Baerveldt implant and Ahmed valve, were subsequently introduced in an effort to improve fluid outflow and offer an alternative to traditional filtration surgery. The EX-PRESS™ glaucoma filtration device (Alcon, Ft. Worth TX), which differs from traditional drainage devices by the lack of an associated end-plate, is an implantable device that is nonvalved and made of medical grade stainless steel. It was initially designed for full thickness scleral implantation, but this method led to sub-optimal results. The surgical technique was later modified so that devices are now implanted under a partial-thickness scleral flap to provide enhanced regulation of fluid flow [2].

Published studies have shown similar IOP-lowering efficacy with use of the EX-PRESS™ device compared to traditional guarded trabeculectomy [3,4]. In addition, the EX-PRESS™ device has also been shown to have lower rates of hypotony and post-operative complications compared to traditional trabeculectomy in some studies [3]. Quicker visual recovery and less post-operative visits were noted in one study in favor of the EX-PRESS™ device when compared to a cohort of patients undergoing trabeculectomy [4].

Despite the building evidence for the surgical utility of the EX-PRESS™ device in some patient populations, little is known about its biocompatibility and how the presence of the stainless steel device influences bleb morphology and local fibrosis [4-7]. The rabbit model for trabeculectomy surgery, which commonly utilizes a silicone tube, has been used extensively to characterize the in vivo response of the eye to various glaucoma implants as well as for the evaluation of different wound modulation agents [8,9]. In the current study, we compare bleb survival, bleb vascularity and local tissue response after implantation of the EX-PRESS™ glaucoma filtration device versus implanted silicone tubes in a rabbit model of filtration surgery.

## Methods

### Study design

All procedures were performed in accordance with The Association for Research in Vision and Ophthalmology Statement for the Use of Animals in Ophthalmic and Vision Research. Twelve New Zealand white rabbits were divided into two groups: filtration surgery with silicone tube implantation (n=6) and filtration surgery with the EX-PRESS™ miniature glaucoma implant. All surgeries were performed by a single surgeon (M.Y.K.) experienced in animal-based ophthalmic surgery.

### Glaucoma filtering surgery technique

The rabbits were anesthesized using intramuscular injections of ketamine and xylazine (ketamine 40 mg/kg; xylazine 20 mg/kg) as well as topical anesthesia (2% lidocaine gel) before initiation of surgery. For the implantation of the silicone drainage device, a fornix based-conjunctival dissection was performed and a 23-gauge needle was used to create a scleral tunnel one millimeter posterior to the limbus for insertion of a 22-gauge cannula (Insyte®; Becton Dickinson Vascular Access, Sandy, UT) into the anterior chamber. The tube was then secured to the scleral bed with 10–0 nylon suture (Ethicon Inc., Somerville, NJ) and efflux of fluid into the subconjunctival space was confirmed. For insertion of the EX-PRESS™ miniature glaucoma implant, a partial-thickness scleral flap was created at the limbus and the implant was inserted through a tract into the anterior chamber created by a 27-gauge needle. Mitomycin-C (MMC) was applied to all eyes following the conjunctival dissection. Two 3×3 mm partial-thickness Weck-cel® spears (Alcon Surgical, Fort Worth, TX) soaked in 0.4 mg/ml MMC were placed over the scleral bed for 5 min before insertion of the cannula or needle. The scleral bed was then irrigated with 40 ml of balanced salt solution (BSS) after removing the soaked spears. Topical moxifloxacin 0.5% (Vigamox®; Alcon) and prednisolone acetate 1% (Predforte®, Allergan, Irvine, CA) were each instilled four times per day for seven days following surgery in all eyes.

### Post-operative evaluation

Daily handheld slit lamp examinations were conducted to document any changes at the surgical site as well as to perform bleb vascularity assessments. Bleb survival was the main outcome measure and defined as the presence of an elevated subconjunctival fluid pocket at the surgical site during slit lamp exam. Central bleb vascularity was graded at three and six weeks post-operatively using a standard scale as noted in Table 1. Anterior segment photographs were obtained weekly at the slit lamp in each eye. A masked independent investigator objectively graded each bleb using slit lamp photographs. All photos were cropped before evaluation of vascularity so that the masked evaluator could not identify which type of tube was in the anterior chamber. Final vascularity grading was based on photos and not slit lamp exams similar to the method used for the Moorfields Bleb Grading System. Bleb survival determination was based on slit lamp exam as photos are not ideal for determining bleb height.

**Table 1:** Mean post-operative vascularity of all groups after three and six weeks of follow-up.

**Group (n=6 each)**	**Three weeks – (SD)**	**Six weeks – (SD)**
Silicone	1.33 (0.95)*	1.55 (1.03)**
EX-PRESS™	1.72 (0.81)*	1.43 (1.72)**

SD=Standard Deviation. *p value of vascularity differences at 3 weeks=0.462. **p value of vascularity differences at 6 weeks=0.886. Grading scale of central bleb vascularity: 0=avascular (>50% avascularity), 1=reduced vascularity (<50% avascular), 2=normal vascularity, 3=increased vascularity (<50% increased vascularity), 4=hypervascularity (>50% increased vascularity).

### Histology

All animals were euthanized at the end of the six-week study. All eyes were then enucleated and immediately immersed in a mixture of 4% paraformaldehyde and 2.5% neutral buffered formalin for 24 h. The globes were dehydrated, embedded in paraffin and sent for microtome sectioning and staining (Hematoxylin and Eosin and Masson Trichrome; Sigma, St. Louis, MO). A modified semi-quantitative grading system to assess cellularity and collagen deposition was used to compare findings between the two groups (Table 2) [10]. Goblet cell number was calculated using the average cell number per high-powered field from six consecutive central bleb cross-sections of each specimen. A prior study has established that normal rabbit conjunctiva typically has about seven goblet cells per high-powered field [11]. A masked evaluation was then performed on all samples. Statistical analysis using one-way ANOVA test was completed for all data sets. A p-value <0.05 was considered statistically significant.

**Table 2:** A comparison of bleb histology between groups.

**Group (n=6 each)**	**Collagen deposition (Masson trichrome)**	**Cellularity (H&E)**	**Mean goblet cell number (SD)**
Silicone	+/−	+/−	1.29 (0.53)*
EX-PRESS™	+/−	+/−	1.15 (0.91)*

*p value for goblet cells between groups p=0.751. -=absent; +/−=weakly present; +,++,+++=present. H&E=Hematoxylin and Eosin. SD=Standard Deviation.

## Results

Histologically, a thin capsule consisting of mild fibroblast proliferation associated with intercellular collagen was present around both implants. Within the area surrounding both implants, a mild infiltration of plasma cells and polymorphonuclear leukocytes was seen. The blebs surrounding both materials were slightly avascular. Both groups were noted to have decreased numbers of goblet cells per high powered field but there was no significant difference between the two groups (p=0.751). No differences in the capsule composition or inflammatory reaction were noted between the two groups (Table 2). Bleb vascularity was similar between both groups at the 3 and 6 week evaluation times (Table 1).

Post-operative bleb survival was similar between the two study groups (Table 3). The average bleb survival for rabbits implanted with the silicone drainage device was 34.30±2.21 days. Rabbits implanted with the EX-PRESS™ miniature glaucoma implant had an average bleb survival of 32.15±4.32 days. This difference was not statistically significant (p=0.303).

**Table 3:** A comparison of post-operative bleb survival between groups.

**Group (n=6 each)**	**Bleb survival days (SD)**
Silicone tube	34.30 (2.21)*
EX-PRESS™	32.15 (4.32)*

*p value of bleb survival between groups=0.303. SD=Standard Deviation.

None of the EX-PRESS™ devices or the 22-gauge cannula were noted to be occluded by tissue at final gross and histological examinations. There were no documented cases of implant extrusion, endophthalmitis, corneal epithelial toxicity, or persistent anterior chamber inflammation in all treated eyes.

## Discussion

Scar formation and fibrosis leading to bleb failure remains a common cause of surgical failure after both trabeculectomy and glaucoma drainage device implantation. Previous studies investigating the histocompatibility of different biomaterials have demonstrated a variable degree of inflammation and fibrosis [5-8,12-14]. In theory, an ideal drainage device would consist of an inert biomaterial with similar or better IOP-lowering capability compared to devices currently in use. The current study compared the histocompatibility of a silicone drainage device, a material commonly used for aqueous drainage devices, and the EX-PRESS™ glaucoma filtration device. Thin fibrotic capsules with intercellular collagen formation and variable amounts of inflammatory cells were noted surrounding both implants. There were no significant differences in biocompatibility between the two materials.

A previously published study looked at the biocompatibility of the EX-PRESS™ device in rabbits. The local tissue reaction at three and six months showed a thin, fibrotic capsule covering approximately 25% of the implant surface area. The capsule and lumen of the device showed no evidence of inflammation [5]. These results are comparable to the histologic findings of our study. However, this study had several limitations including; 1) Antifibrotic agents were not used at the time of surgery 2) The protocol did not include use of a scleral flap for EX-PRESS™ implantation which is now a standard procedure and 3) The conjunctiva was not sutured at the conclusion of surgery and the authors did not use post-operative drops as is typical of modern day filtration surgery. These limitations were addressed in the current study and allow for a direct comparison of in vivo findings in a way that is more in line with modern day use of the EX-PRESS™ device.

The histopathology of the EX-PRESS™ shunt has also been studied after implantation in human eyes [6,7]. Aziz et al. [6] reported on the histopathologic features of an enucleated eye in an eighty-six year-old male who had previously undergone EX-PRESS™ implantation for neovascular glaucoma. Examination revealed minimal cellular reaction surrounding the implant. A thin fibrocellular tissue layer was present beneath the implant without evidence of granulomatous inflammation or marked cellular inflammatory infiltrate. De Feo et al. [7] looked at the tissue reaction surrounding the EX-PRESS™ implant in a seventy-three year-old female diagnosed with uncontrolled primary open-angle glaucoma. They found a small shoulder of fibrous tissue between the spur and flange of the device. The remainder of the device was covered by a thin, fibrous capsule, but no inflammatory cells were found within the capsule.

Prior studies evaluating the biocompatibility of implanted materials have demonstrated a more intense inflammatory reaction compared to our findings and others. Ayyala et al. [8] looked at the inflammatory reaction associated with the Ahmed polypropylene device compared to the Baerveldt silicone device in a rabbit model. Inflammatory cells were found in the fibrous capsule of both implants. The Baerveldt silicone device was associated with a lower amount of inflammation compared to the polypropylene version of the Ahmed valve. A follow-up study demonstrated that the polypropylene of the Molteno device was more inflammatory than the Krupin silicone device [12]. Histopathologic evaluation revealed a fibrous capsule containing inflammatory cells surrounding both biomaterials with the Molteno polypropylene demonstrating a higher grade of inflammation and fibrosis.

Our study does provide useful information regarding the histocompatibility of silicone versus the stainless steel of the EX-PRESS™ however there are some limitations. First, this study was performed in a well established rabbit filtration surgery model and the results may not directly correlate with findings in humans [5,8,12,13]. Second, we did not include a control group without MMC application to evaluate the influence of the antifibrotic agent or its potential alteration of effect from the material itself. The use of MMC in this rabbit model of glaucoma filtration surgery can lead to loss of goblet cells, a decrease in fibroblast proliferation as well as a significant increase in bleb survival. For this reason, the same dose of MMC was used in both groups to remove any potential for confounding variables. The study was designed to closely simulate current day practice, which typically includes use of MMC. The results of this study suggest that the stainless steel EX-PRESS™ drainage device has similar biocompatibility to silicone implants in the eye. Both devices resulted in functioning blebs of similar clinical characteristics and survival time, as well as comparable histological findings. The presence of medical grade stainless does not seem to negatively alter the healing response after EX-PRESS™ implantation compared to previously accepted glaucoma device materials such as silicone. Further studies are needed to determine if these results are consistent in humans over long-term follow up.

## References

[1] Quigley HA, Broman AT (2006). The number of people with glaucoma worldwide in 2010 and 2020.. Br J Ophthalmol.

[2] Hendrick AM, Kahook MY (2008). Ex-PRESS mini glaucoma shunt: surgical technique and review of clinical experience.. Expert Rev Med Devices.

[3] Maris PJ, Ishida K, Netland PA (2007). Comparison of trabeculectomy with Ex-PRESS miniature glaucoma device implanted under scleral flap.. J Glaucoma.

[4] Good TJ, Kahook MY (2011). Assessment of bleb morphologic features and postoperative outcomes after Ex-PRESS drainage device implantation versus trabeculectomy.. Am J Ophthalmol.

[5] Nyska A, Glovinsky Y, Belkin M, Epstein Y (2003). Biocompatibility of the Ex-PRESS miniature glaucoma drainage implant.. J Glaucoma.

[6] Aziz H, Fantes F, Dubovy S. (2011). Histopathology of the Ex-PRESS Shunt.. Ophthalmic Surg Lasers Imaging.

[7] De Feo F, Jacobson S, Nyska A, Pagani P, Traverso CE (2009). Histological biocompatibility of a stainless steel miniature glaucoma drainage device in humans: a case report.. Toxicol Pathol.

[8] Ayyala RS, Harman LE, Michelini-Norris B, Ondrovic LE, Haller E, Margo CE, Stevens SX (1999). Comparison of different biomaterials for glaucoma drainage devices.. Arch Ophthalmol.

[9] Sherwood MB. A sequential, multiple-treatment, targeted approach to reduce wound healing and failure of glaucoma filtration surgery in a rabbit model (an American Ophthalmological Society thesis). Trans Am Ophthalmol Soc. 2006;104:478–92. PMC1809919 17471357

[10] Hackett R, McDonald T, eds. Dermatoxicology. Washington D.C.: Hemisphere Publishing; 1996.

[11] Kahook MY, Noecker R (2008). Quantitative analysis of conjunctival goblet cells after chronic application of topical drops.. Adv Ther.

[12] Ayyala RS, Michelini-Norris B, Flores A, Haller E, Margo CE (2000). Comparison of different biomaterials for glaucoma drainage devices: part 2.. Arch Ophthalmol.

[13] Lloyd MA, Baerveldt G, Nguyen QH, Minckler DS (1996). Long-term histologic studies of the Baerveldt implant in a rabbit model.. J Glaucoma.

[14] Loeffler KU, Jay JL (1988). Tissue response to aqueous drainage in a functioning Molteno implant.. Br J Ophthalmol.

